# Synergistic effect of sunlight induced photothermal conversion and H_2_O_2_ release based on hybridized tungsten oxide gel for cancer inhibition

**DOI:** 10.1038/srep35876

**Published:** 2016-10-24

**Authors:** Cong Wang, Yibo Gao, Xinghua Gao, Hua Wang, Jingxuan Tian, Li Wang, Bingpu Zhou, Ziran Ye, Jun Wan, Weijia Wen

**Affiliations:** 1 Institute of Microstructure and Property of Advanced Materials, Beijing University of Technology, Beijing 100124, China; 2 Department of Physics, The Hong Kong University of Science and Technology, Clear water bay, Kowloon, Hong Kong; 3 Division of Environmental Science, The Hong Kong University of Science and Technology, Clear water bay, Kowloon, Hong Kong; 4 Shenzhen PKU-HKUST Medical Center, Biomedical Research Institute, Shenzhen-PKU-HKUST Medical Center, Shenzhen, China; 5 Materials Genome Institute, Shanghai University, Shanghai 200444, China; 6 Department of Laboratory Medicine, Renji Hospital, School of Medicine, Shanghai Jiaotong University, Shanghai, China; 7 Institute of Applied Physics and Materials Engineering, University of Macau, Taipa, Macau

## Abstract

A highly efficient photochromic hydrogel was successfully fabricated via casting precursor, which is based on amorphous tungsten oxide and poly (ethylene oxide)-*block*-poly (propylene oxide)-*block*-poly (ethylene oxide). Under simulated solar illumination, the hydrogel has a rapid and controlled temperature increasing ratio as its coloration degree. Localized electrons in the amorphous tungsten oxide play a vital role in absorption over a broad range of wavelengths from 400 nm to 1100 nm, encompassing the entire visible light and infrared regions in the solar spectrum. More importantly, the material exhibits sustainable released H_2_O_2_ induced by localized electrons, which has a synergistic effect with the rapid surface temperature increase. The amount of H_2_O_2_ released by each film can be tuned by the light irradiation, and the film coloration can indicate the degree of oxidative stress. The ability of the H_2_O_2_-releasing gels *in vitro* study was investigated to induce apoptosis in melanoma tumor cells and NIH 3T3 fibroblasts. The *in vivo* experimental results indicate that these gels have a greater healing effect than the control in the early stages of tumor formation.

Photoresponsive functional composites have aroused worldwide research interests recently due to their enhancing photoelectrochemistry^1^,^2^,^3^, light-harvesting^4^ and biomedical applications^5^. Among the numerous composites, materials based on transition metal oxides (TMOs) exhibits desirable behaviors, and TMOs have been devoted great attention owing to high-temperature superconductivity^6^, colossal magnetoresistance^7^ and controllable charge carrier density^8^. The tunable localized surface plasmon resonance (LSPR) of non-stoichiometric tungsten oxide has been shown to exhibit potential for use in light-harvesting, bioimaging and sensing applications^8^. The potentially practical distinctive and controlled LSPR effects can be switched on and off in response to various stimuli, such as temperature, pressure and irradiation. Although noble metals LSPR is already widely used in photothermal therapy (PTT)^9^,^10^, it is needed to exploit more and more applied LSPR in PTT to reach better controllability and therapy. Although the wide success of noble metal LSPR was applied in photothermal therapy, it is still needed to exploit alternative materials with chemical stability and low cost^11^. Hybridization strategy enables a nanoscale photosensitizer to achieve an excited state from which it releases vibrational energy due to their less energetic, relatively high charge carrier densities and lower toxicities to cells and tissues^12^,^13^,^14^. In the past two years, the application of TMOs in PTT has received increasing attention on controllable therapies^15^,^16^,^17^. Although the quality factors of TMOs are less than those of gold and silver metal, the localized electrons in TMOs are always involved in the coloration process or infrared absorbance, which might impact their effectiveness in PTT. Thus, the influence of localized electrons on these processes should be investigated at a fundamental level and in medical applications to solve academic and technical problems.

Tungsten oxide exhibits a strong color change when small ions such as Li^+^ or H^+^ are intercalated into the structure or when the structure is oxygen deficient^18^. Understanding the localized electron mode in WO_3−x_ will enable the TMO nanostructure to be optimally designed for bioimaging, sensing and light-harvesting applications. However, the localized electron density in nanoparticles still limits their practical use. In photo induction processes, amorphous tungsten oxide (a-WO_3_) has been shown to respond quickly and efficiently, and it has good reversibility and high optical density^19^,^20^,^21^. The localized electrons in a-WO_3_ can be controlled to achieve switchable and tunable photoresponsiveness because the a-WO_3_ absorption spectrum has been demonstrated to be tunable in the visible light and infrared regions.

In addition, photogenerated radicals can induce tumor apoptosis with minimum cytotoxicity^22^. Many TMOs that could be used in nanomedicine exhibit anti-cancer activities that might be due to oxidative stress processes^23^. Several research groups reported that when TiO_2_ is irradiated with UV light, hydroxyl radicals (•OH), which are a type of reactive oxygen species (ROS), are generated, inducing oxidative stress^24^. In particular, TiO_2_ nanoparticles with large surface to volume ratios have been reported to be 100 times more toxic to human fibroblast and lung epithelial cells than an equivalent sample composed mostly of rutile TiO_2_^24^. However, the synergistic effects of radical generation and localized surface plasmons are rarely discussed. Because ROS can damage cells and tissues cannot be widely used *in vivo*, materials that allow for controlled H_2_O_2_ release are urgently needed for quantitative treatments in various fields.

In this work, to achieve the aforementioned goals, a flexible, topological a-WO_3_ nanocomposite that exhibits efficient photochromic behavior was condensed from a precursor whose synthesis was previously described^18^. To simulate the ambient environment, the material was irradiated by a Xenon lamp that emits the entire spectrum from the UV to infrared regions. Because the material is highly sensitive to weak room light irradiation, it generates H_2_O_2_, which can inhibit the proliferation of metastatic melanoma cells, during the irradiation process. Furthermore, the amount of generated H_2_O_2_ is highly influenced by the temperature used to heat the a-WO_3_ surface due to the high photothermal absorbance of a-WO_3_, which also impacts the peroxide diffusion through polydimethylsiloxane (PDMS)^25^. In previous work^14^, this photochromic material not only prevents photoinduction by weak room light, but it also provides a reversible, controllable oxidative stress in the extracellular matrix caused by the sustainable, quantitative release of H_2_O_2_. Here, the synergistic effect of the H_2_O_2_ generation and the photothermal effect induced by the localized electrons are studied to enhance cancer cell apoptosis *in vitro* and *vivo* experiments. It is important for maintaining the appropriate growth, development, and death of healthy cells at the required times^26^,^27^,^28^,^29^,^30^. By transferring the photoelectrochemical ability of the framework to the ROS, the H_2_O_2_ concentration and release rate can be controlled by the selected hole scavenger. Because the fabricated curative film exhibits a greater therapeutic effect on tumor cells than the control, it has potential clinical applications in PTT, and the selected hole-scavenger PT building block could be used in a broader range of potential applications in H_2_O_2_-dependent tissues and regenerative engineering.

## Results and Discussion

### Photothermal effect induced by resonance over entire solar spectrum

**Figure 1:**
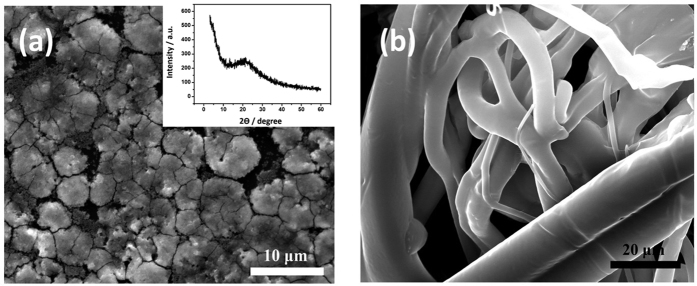
The morphologies of (**a**) a-WO_3_ and its XRD pattern in inserted figure, (**b**) fibers substrate in SEM images.

The characterization of nanocomposite in aqueous state and oxygen radical release has been studied previously^14^,^18^. Herein, the photothermal technology of hydrogel upon solar irradiation is mainly discussed. Compared with other functionalized hole scavengers, sulfate-functionalized a-WO_3_ is advantageous because of its greater electron-hole separation attributed to its strong Brønsted acid sites. The highly polarized state of the acidic surface is favorable for photohole trapping, which contributes to a considerably enhanced quantum yield of oxygen radicals. The release of them happen on the surface of a-WO_3_, where the morphology of a-WO_3_ is evaluated and shown in SEM image (Fig. 1a), there were uniform and homogeneous distribution sheets of complex, which were achieved successfully by mixing the high molecule weight surfactant with tungstate. The XRD is shown in inserted figure. The hybrid film was fabricated by casting precursor onto fibers or gauze, as shown in Fig. 1b, a-WO_3_ was distributed uniformly on the surface of fibers. The unconsolidated fiber is also benefit for the oxygen gas diffusion.

**Figure 2:**
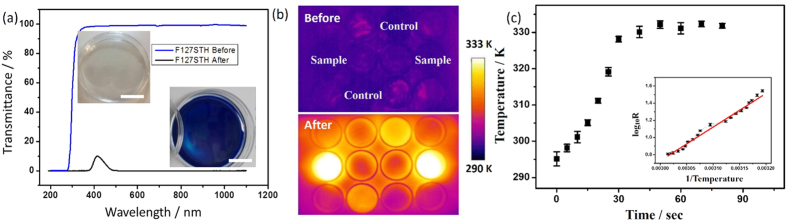
(**a**) Recorded transmittance of the casted hybrid nanocomposite before and after UV irradiation measured by UV-Vis spectroscopy, the scale bars of sample images are 2.7 cm; (**b**) IR thermal image of a 12-well plate containing samples, controls and blanks before and after 5 mins of irradiation; (**c**) Temperature increase dependence on irradiation time by AM 1.5 G, resistance analysis shown as inserted curve.

After the water was evaporated, a flexible hydrogel film was formed, and its optical absorption was measured (Fig. 2a). The reduced film absorbs nearly the entire spectrum from UV_b_ to the near infrared region, which is in sharp contrast to the absorption of the nanocomposite before irradiation. The entire spectrum including UV_a_ and UV_b_ is beneficial for inhibiting the growth of melanoma cells. More importantly, solar irradiation in this range is effectively used during bleaching because the solar energy is satisfactorily converted into heat energy.

An infrared thermographic camera was used to monitor the a-WO_3_ surface temperature before and after irradiation. It can be visualized as Fig. 2b. Compared with blank and controlled (without a-WO_3_) wells, the sample one reach over 55–65 °C quickly. The film surface temperature quickly increases to approximately 60 °C and remains at this temperature for as long as 5 mins under ambient conditions, indicating that the hybrid a-WO_3_ surface heat increase are much more efficient than the their individual components, so that they might provide equivalent heating at reduced dosage, alleviating potential side effects. The final temperature increase along irradiation, as shown in Fig. 2c, can reach 60 °C in a short time. The lgR value is direct proportional to 1/T in inserted curve indicated that the film material is semiconductor-like hydrogel. Sustainable oxygen release should occur at air-solid interface. Thus, the sufficient heating energy generated by the localized electrons could play a role in both the bleaching process^31^ and the H_2_O_2_ generation. If the film is thin, its coloration-bleaching cycle efficiency will increase significantly.

### Sustainable and controlled H_2_O_2_ release

**Figure 3:**
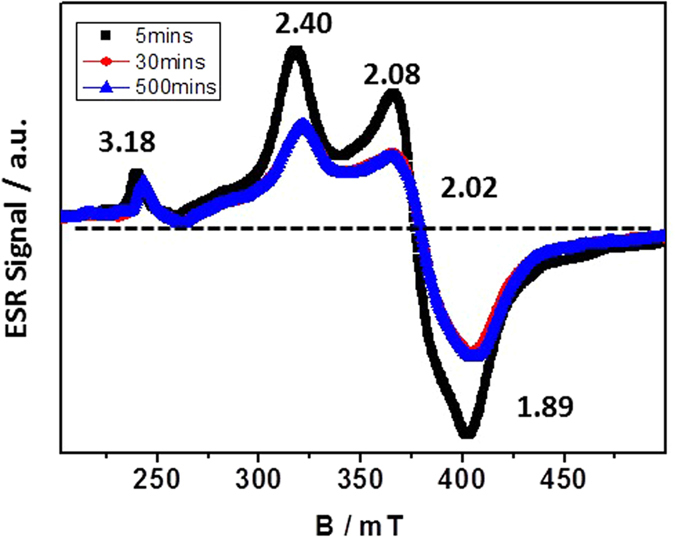
Electron spin resonance spectra of gel at 77 K under vacuum conditions after induction for 5 mins, 30 mins and 500 mins.

**Figure 4:**
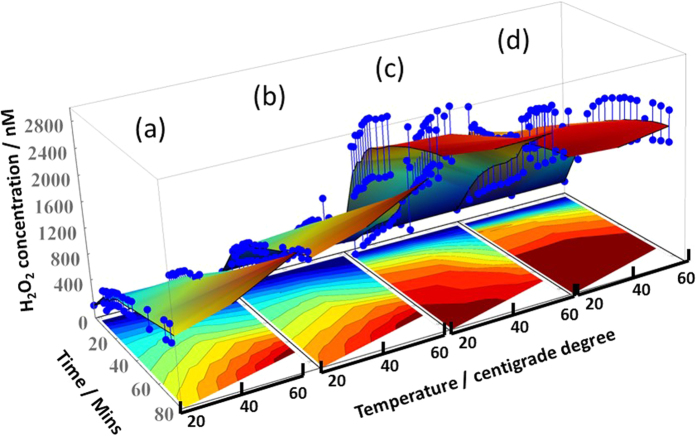
Accumulation of H_2_O_2_ concentration obtained over time and various temperature after UV irradiation for 30 secs under cold (**a,b**) and heat (**c,d**) surroundings, respectively. The original solution precursor was diluted into 2 and 8 times for fluorescence study.

To study oxygen radical evolution at low temperatures, the ESR spectra of the samples were acquired at 77 K under vacuum conditions after different induction times (Fig. 3). For the Brønsted sites, three broad peaks appear upon UV irradiation, which is consistent with the generation of reductive electrons by the multivalent tungstate. The different Brønsted acid peaks are observed due to their weak coordination to the ROS generators at g = 3.18 and 1.89. The signal at g = 1.89 is assigned to surface holes or subsurface cationic sites, whereas the peak at g = 3.18 is enhanced by the presence of acidic anions. The peaks at g = 2.40 and 2.02 are caused by the reaction of a superoxide to form H_2_O_2_. The ROS signal increases over the first 5 mins and then slowly decreases until it finally disappears. However, when this signal is induced and trapped by stable free radical agents, 2,2,6,6-tetramethy-1-piperidinyloxy free radical (TEMPO) at low temperatures, it is still observed after 500 minutes, and the sample remains blue in color after being removed from the 77 K chamber. As shown in Fig. 4, the quantitative peroxide measurements by fluorescence give striking results. The coloration and bleaching processes are shown as a function of time in Eqs 1 and 2 and Eqs 3 and 4, respectively. After the electron and hole are separated, the photogenerated hole splits water to produce a hydroxyl radical, which eventually forms stable hydrogen peroxide. During the charge transfer, an electron spin transition occurs simultaneously with the polarization of the long-term W_5d_-O_2p_ radical triplet according to the Boltzmann distribution. The separated electron can then react with oxygen to form 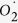, which can accept hydrogen ions to generate H_2_O_2_. Thus, in this work, the intensity of the optical absorption peak due to the electron-hole separation might be proportional to the oxidative stress caused by the sustainable H_2_O_2_ release. This biomaterial can be utilized for quantitative oxidative studies based on optical spectroscopy.

**Figure 5:**
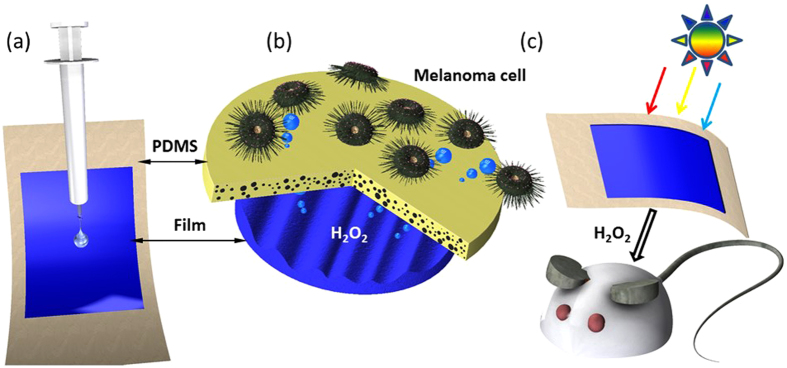
(**a**) Facile casting of the precursor on the fiber substrate; (**b**) PDMS-packaged hybrid film; (**c**) tunable, controlled H_2_O_2_ release over the entire white light spectrum.

The sample with the PDMS layer through which H_2_O_2_ could diffuse was used for the fluorescence measurements and cell studies. As shown in Fig. 5a, the hybrid film was fabricated by casting precursor onto fibers or gauze as shown in Fig. 1b. The cell behavior and *in vivo* study method are illustrated in Fig. 5b,c, respectively. The precursor was easily casted onto commercial fibers, and the nanocomposite strongly adhered to the fibers during water evaporation. PDMS was chosen due to its biocompatibility and cell adaptability. The diluted species were transported through the PDMS layer. This membrane diffusion process, which is analogous to diffusion through skin in the *in vivo* study, was studied by monitoring the H_2_O_2_ fluorescence intensity.


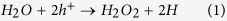



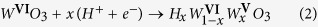



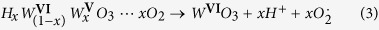



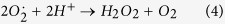


A precise, quantitative controlled H_2_O_2_ release study was performed using hydrogel with various tungstate concentrations and at different temperatures. It was assumed that irradiation with a 153 mW/cm^2^ Xenon lamp for 30 secs was adequate for H_2_O_2_ generation. The unheated sample was placed on an ice pack. A relatively small concentration compared to those detected between 10 and 80 min during the calibration was used to predict the total H_2_O_2_ release concentration. H_2_O_2_ was detected using fluorescent resorufin. The use of H_2_O_2_-sensitive Amplex^®^ Red results in an increase in the fluorescence curve within 5 minutes after irradiation. The effects of the tungstate concentration and surrounding temperature on the amount of H_2_O_2_ released were determined as shown in Fig. 4. The original solution sample was diluted by 2 and 8-fold. For a given cold or heat surroundings, the H_2_O_2_ release efficiency of this flexible hybrid film is considerably enhanced when the lamp is fully lit and heated. The detected H_2_O_2_ concentrations increase with concentration of tungstate and decrease without under high-temperature Xenon lamp. The amount of H_2_O_2_ released can be controlled by changing the concentration of the “a-WO_3_ drug”. More importantly, the photothermal effect gave an impact on H_2_O_2_ generation in this condition.

### Photothermal therapy on Melanoma cell apoptosis and *in vivo* experiments

Reversible, sustainable controlled H_2_O_2_ release has potential for clinical application in cancer inhibition, cytological behavior studies and environmental decontamination. The localized electrons dominated the H_2_O_2_ release can be measured precisely on various ambient temperature. Accordingly, the optimal photochromic a-WO_3_ was chosen for the *in vitro* and *in vivo* experiments.

Under irradiation by the entire solar spectrum, ROS are sustainably released from the photochromic a-WO_3_. The long-term, tunable H_2_O_2_ release might induce tunable oxidative stress to tumor cells. Selective oxidative stress is most suitable for tumor inhibition therapy. Besides that, the solar spectrum also gave additional heat energy for enhanced tumor inhibition.

**Figure 6:**
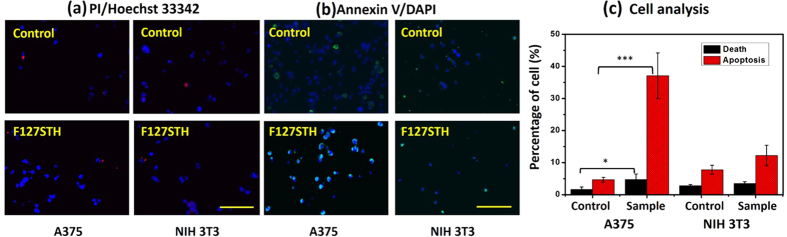
A375 cell behavior on PDMS (control), the PDMS-packaged sample, and PDMS dosed with 1 μM A.R. H_2_O_2_ or 1 μg/mL doxorubicin after treatment. (**a**) Fluorescence images showing cell death by PI staining (red); (**b**) fluorescence images showing cell apoptosis by Annexin V staining (green); (**c**) histogram of the percentages of dead and apoptotic cells. All scale bars are 200 μm. The cell nuclei were stained by Hoechst 33342 or DAPI (blue). p < 0.05*; p < 0.005***.

The surface temperature of the tumors mice reached 50–60 °C after laser irradiation, in contrast to tiny increase for irradiated tumors on untreated mice. As shown in Fig. 5a,b, the fluorescence images indicate that the radicals can be transformed into H_2_O_2_, which can cause oxidative stress that leads to a series of events, including DNA damage and apoptosis. To determine whether the studied anti-tumor materials can induce cell apoptosis by oxidative stress and not be harmful to normal cells, a *in vitro* cell apoptosis analysis (Fig. 6a,b) was performed in which the location of phosphatidylserine (PS) on cell membrane was stained by Annexin V. Compared to the untreated group, melanoma cell was induced into apoptosis significantly. In contrast, there is no obvious harm for Epithelium cell (NIH/3T3). The statistical result was showed as Fig. 6c. With drug treatment, the A375 cell showed significant apoptosis percentage compared to the control group while no significant apoptosis change was observed in the NIH/3T3 group. Further, we found that our F127 STH mainly induce the cell apoptosis other than cell death.

**Figure 7:**
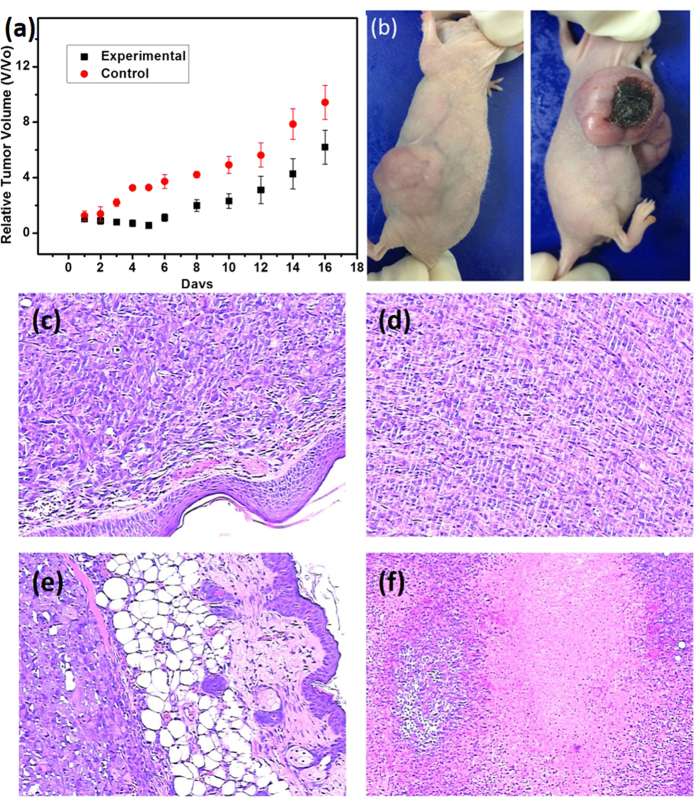
(**a**) Tumor therapies by hydrogel as a function of time. Tumor growth curves of different groups after treatment. The tumor volumes were normalized to their initial sizes. (**b**) Photographs of the experimental (left) and control (right) sides of one mouse after nanomedicine treatment. (**c–f**) HE staining of tumor tissue of mice in one week.

Furthermore, the photodynamic effect of the optimal hydrogel formulation was determined and employed the *in vivo* study. Hairless tumor model mice with a tumor volume of 70 mm^3^ were topically treated with 1 ml gel to smear on the skin. The volume of skin cancer treated nothing increased over time regardless of laser irradiation (Fig. 7a,b). Only one side of the cancerous skin was treated with irradiated gel, whereas the other side of the skin was not treated for comparison. In the case of treatment, the tumor volume was significantly suppressed in the presence of after light irradiation by the photodynamic effect. For the H_2_O_2_ release group, the percent of the drug agent coated relative to the tumor tissue volume were smaller than the control group particularly in the early stages of treatment. Then, the level of inhibition is lower than the previous one after 7 days. It is suggested that the small concentration H_2_O_2_ had a synergistic effect with photothermal process, which might enhance the curative effect at the earlier tumor treatment times.

Further evidence of anti-tumor effect was provided by H&E staining as shown in Fig. 7c–f. Cell nuclei stained blue, and intracellular and extracellular proteins stained pink. Morphological assessments were made in relation to treatment with gel gauze within 2 weeks in A375 tumors. As controls group shown, A375 tumors treated without gel showed their usual histological appearance of poor differentiation and limited necrosis (Fig. 7c,d). In contrast, 7-days sample treated with gel under exposure induced obvious tissue necrosis to different extents in A375 tumors (Fig. 7e,f), Pyknosis was observed in tumor tissues after treatment with F127STH in the presence of light irradiation, reflecting necrosis or apoptosis of tumor cells. Pyknosis is the irreversible condensation of chromatin in the nucleus of a cell, usually associated with necrosis or apoptosis^32^. 1-week treatment caused local and limited necrosis but accompanied with manifest haemorrhage. However, further treatment might not be able to efficiently suppress tumor growth, which may be related to drug dose.

The method presented herein might provide a way to generate the optimal H_2_O_2_ concentration to allow its diffusion into the skin. The localized electrons act as small polarons that exhibit resonance over a broad wavelength range from blue light to the infrared region. As the bleaching time increases, the absorbed energy can eventually be converted into heat energy. A tumor-dependent pore cutoff size ranging from 200 nm to 2 mm, which might allow H_2_O_2_ molecules to access malignant tumor cells, was determined by direct observations of tumor vasculatures^33^,^34^. Therefore, the remarkable inhibition and curative properties of the nanocomposite gel in the early stages shown in Fig. 7 are not surprising. The cumulative H_2_O_2_ concentration is as high as several micromoles in the experimental system, which might have increased the effective concentration of peroxide acting as a nanomedicine. In addition to the effective therapy in the tumor tissues observed in this study, the sustained release of H_2_O_2_ during long-term treatment might suppress tumor growth over the entire 3-week therapy treatment.

## Conclusions

Photochromic composite hydrogel was successfully fabricated based on anti-bacteria polymer and amorphous tungsten oxide. After dehydration, the material exhibits a fast coloration response along with rapid temperature increase upon solar irradiation. The enhanced photo-thermal effect was associated with the released H_2_O_2_ in the whole coloration process, which was mainly achieved by localized electrons. Moreover, the ability of the H_2_O_2_-releasing nanocomposite to induce apoptosis in melanoma tumor cells was investigated, and the *in vivo* experimental results indicate that these nanocomposites have a greater healing effect than the control in the early stages of tumor formation.

## Methods

### Preparation of composite film

Composite mixture solution was prepared as previous work^13^. The gel was condensed by solution F127STH, which contains pluronic F127, sodium tungstate and sulfuric acid (weight ratio 20:1.3:0.5). The precursor was casted on commercial PE fiber after plasma cleaning. The resulting hydrogel was aged and dried in air for about 1 day, giving the hybrid film with 40% of precursor content in weight. A PDMS thin-film layer (~100 μm) was spin-coated on the hybrid film, effectively immobilizing the structural sample at the bottom of the culture dish. This configuration allowed sunlight to pass through the PDMS film to the melanoma cell-containing medium on top of the PDMS layer.

### Cell study and *in vivo* experiments

A Xenon lamp was selected as the radiation source, and all of the films for cell analysis were overexposed to UV light for 30 min before the cell experiments. The hydrogel sample used for cell studies is named F127STH. The human melanoma A375 cell line and mice NIH 3T3 fibroblasts were obtained from ATCC. The cells were maintained in Dulbecco’s modified Eagle’s medium with 4500 mg/L glucose (HG-DMEM) and supplemented with 10% fetal bovine serum (FBS), 100 U/mL penicillin and 100 μg/mL streptomycin in a humidified atmosphere at 37 °C with 5% CO_2_. The HG-DMEM, FBS, penicillin and streptomycin were purchased from GIBCO Invitrogen.

The cells were seeded onto glass slides at a density of 1.0 × 10^5^ cells/cm^2^ and incubated for 24 h before introducing the hydrogel films. The cells were not exposed to UV light. After the cells were treated with the hybrid films for 24 h, the cell ROS, apoptosis and death were determined.

The CellROX Deep Red Reagent (Life Technologies) was used to detect intracellular ROS in the live cells. After stimulation by adding the probe to the complete medium and incubating at 37 °C for 30 min, the cells were stained with 5 μM CellROX Deep Red Reagent and Hoechst 33342. To detect cell apoptosis, phosphatidylserine (PS) staining with Alexa Fluor 488 Annexin V (Life Technologies) was performed. In apoptotic cells, PS is translocated from the inner to the outer leaflet of the plasma membrane where it is exposed to the external cellular environment and can be detected by Annexin V.

The cells were visualized with an Olympus fluorescence microscope (Olympus IX 71, Japan). The immunofluorescence data were quantified using the Image-Pro Plus 6.0 software (Media Cybernetics, Silver Spring, MD, USA) and statistically analyzed using Student’s t-test, in which p values of 0.05 were considered statistically significant.

All animals were treated according to the Guide for the Care and Use of Laboratory Animals of the National Institutes of Health. The protocol was approved by the Committee on the Ethics of Animal Experiments of the Shenzhen-PKU-HKUST Medical Center (Permit Number:158). Melanoma A375 tumors were induced in 8-week-old BALB/c nude mice. The mice were injected subcutaneously in the left and right flanks with approximately 3 × 10^6^ melanoma cells suspended in 100 μL of phosphate-buffered saline (PBS). During the drug delivery and observation period, the mice were exposed to a 35 w Am 1.5 Xenon Lamp. Tumor-targeting studies were conducted after the mice developed tumors larger than 100 mm^2^. The mice were divided into groups of 6 to minimize the influence of variations in the tumor size among the groups. Within 21 days, the mice had grown tumors, and the tumor sizes of the mice dosed with the controlled H_2_O_2_ release gel and of the control mice were recorded. Each drug was dehydrogenated in 2 mL nanocomposite after 12 h at 65 °C, and the resulting unguent gel on the polyethylene fibers was spread on the skin outside the tumor once every 24 h. To determine the fully dose, gauze was included along with the tissues above the formulation vials. The data are presented as the means and standard deviations of the percent of injected dose per gram of tissue unless otherwise noted. After 7 days, one group mice were killed, their tissue were removed and paraffin-embedded. Experimental and controlled sections were H&E-stained.

### Characterization

UV-Vis transmittance spectra were measured from 200 to 1100 nm. Heating curves were recorded by an IR thermal camera (FLIR system, ThermaCAM Researcher). The film coloration and bleaching data were recorded under irradiation by a simulated solar AM1.5G lamp with an average irradiance of 153 mW/cm^2^. The cold environment is created by ice pack. The electron spin resonance (ESR) spectra were recorded at 77 K using a JEOL JES-FA200 spectrometer. Scanning electron microscopy (SEM) images were obtained with a JEOL 6390 scanning electron microscope. The SEM samples were prepared by sintering sample powder onto a Si substrate. The amount of H_2_O_2_ was determined from the fluorescence intensity of oxidized Amplex^®^ Red (10-acetyl-3,7-dihydroxyphenoxazine). The Amplex^®^ Red reagent reacts with H_2_O_2_ in the presence of horseradish peroxidase (HRP) in a 1:1 stoichiometric ratio to form fluorescent resorufin. Here, this method was used to quantify the amount of hydrogen peroxide desorbed from various samples. The resorufin absorption was measured in a 96-well plate at 571 nm with a UV/Vis spectrophotometer (Perkin Elmer, Lambda 35). The Amplex^®^ Red Catalase Assay Kit was purchased from Molecular Probes (USA). For the calibration curve, the absorption data collected at 571 nm over a wide H_2_O_2_ concentration images were created by Mathematic software.

## Additional Information

**How to cite this article**: Wang, C. *et al.* Synergistic effect of sunlight induced photothermal conversion and H_2_O_2_ release based on hybridized tungsten oxide gel for cancer inhibition. *Sci. Rep.* **6**, 35876; doi: 10.1038/srep35876 (2016).
